# Shared clinical decision-making experiences in nursing: a qualitative study

**DOI:** 10.1186/s12912-021-00597-0

**Published:** 2021-06-01

**Authors:** Fen-Fang Chung, Pao-Yu Wang, Shu-Chuan Lin, Yu-Hsia Lee, Hon-Yen Wu, Mei-Hsiang Lin

**Affiliations:** 1 Department of Nursing, Chang Gung University of Science and Technology, Taoyuan, Taiwan, R.O.C.; 2 Department of Nursing, MacKay Junior College of Medicine, Nursing, and Management, New Taipei City, Taiwan, R.O.C.; 3 Department of Nursing, Mackay Memorial Hospital, Taipei, Taiwan, R.O.C.; 4 Department of Internal Medicine, Far Eastern Memorial Hospital, New Taipei City, Taiwan, R.O.C.; 5 Institute of Epidemiology and Preventive Medicine, College of Public Health, National Taiwan University, Taipei, Taiwan, R.O.C.; 6 Department of Internal Medicine, National Taiwan University Hospital and College of Medicine, Taipei, Taiwan, R.O.C.; 7 School of Medicine, College of Medicine, National Yang Ming Chiao Tung University, Taipei, Taiwan, R.O.C.; 8 School of Nursing, National Taipei University of Nursing and Health Sciences, No. 365, Mingde 1st Rd. Beitou Dist, Taipei, Taiwan, R.O.C.

**Keywords:** shared decision making, nurses, in-service education, SDM

## Abstract

**Background:**

Shared decision making (SDM) is a patient-centered nursing concept that emphasizes the autonomy of patients. SDM is a co-operative process that involves information exchange and communication between medical staff and patients for making treatment decisions. In this study, we explored the experiences of clinical nursing staff participating in SDM.

**Methods:**

This study adopted a qualitative research design. Semistructured interviews were conducted with 21 nurses at a medical center in northern Taiwan. All interview recordings were transcribed verbatim. Content analysis was performed to analyze the data.

**Results:**

The findings yielded the following three themes covering seven categories: knowledge regarding SDM, trigger discussion and coordination, and respect of sociocultural factors.

**Conclusions:**

The results of this study describe the experiences of clinical nursing staff participating in SDM and can be used as a reference for nursing education and nursing administrative supervisors wishing to plan and enhance professional nursing SDM in nursing education.

**Supplementary Information:**

The online version contains supplementary material available at 10.1186/s12912-021-00597-0.

## Background

Shared decision making (SDM) is increasingly advocated as the preferred model to engage patients in making decisions regarding their diagnosis, treatment, or follow-up when more than one medically reasonable option is available [1]. SDM is a patient-centered medical care service model that emphasizes the provision of high-quality patient-based clinical care and focuses on improving patient satisfaction [2, 3]. SDM is defined as “an approach where clinicians and patients share the best available evidence when faced with the task of making decisions, and where patients are supported to consider options, to achieve informed preferences” [4]. Various models have been developed to demonstrate how SDM can be applied in the clinical setting [5]. Elwyn et al. [4] proposed a model demonstrating the application of SDM in clinical practice; this model was based on three key steps: choice talk, option talk, and decision talk. The essential elements of SDM are as follows: (1) defining or explaining the problem, (2) evaluating available options, (3) discussing the advantages and disadvantages of those options, (4) clarifying the patient’s values and preferences, (5) discussing the patient’s ability or self-efficacy, (6) discussing health care professionals’ knowledge or recommendations, (7) checking and clarifying the patient’s understanding; (9) making or explicitly deferring a decision; and (9) arranging a follow-up [5]. In recent years, the centrality of the patient’s voice in SDM has been increasingly discussed [6]. Mathijssen et al. [7] investigated the SDM-related knowledge, attitude, and experience of 147 medical staff in the field of rheumatism and indicated that enhancing medical care professionals’ understanding of SDM concepts is the critical first step for improving the application of SDM in clinical practice.

The literature on SDM is extensive. Studies have described the individual components of SDM including facilitators and barriers to the achievement of SDM [1, 8]. In the nursing literature, SDM is discussed from an evidence-based practice perspective [9] and the practice perspective of critical nurse–patient interaction [10]. Nurses who participate in SDM can more effectively control their practice and have higher job satisfaction; moreover, hospitals that adopt SDM can improve patient care [11]. Nursing staff are the essential members of a medical team; their participation in the SDM process as well as their understanding of basic concepts and principles related to the decision-making process are particularly crucial [9, 12]. Tariman et al. [13] investigated the role of nursing staff in the SDM process for cancer care and reported that nursing staff play different roles under differing time points and environments in the cancer SDM process; these roles include health educator, spokesperson, data collector, symptom and side-effect handler, information sharer, and psychological supporter.

Jo, An, and Lee [14] indicated that SDM is a comprehensive concept based on the values and autonomy of patients, family members, doctors, and nursing staff and involves the sharing of information regarding treatment options and decision-making methods. In addition, these partnerships may extend into a large network including family members and other professionals or nonprofessional community organizations. This collective involvement further compounds the decision-making process [15]. Elwyn et al. [4] indicated that low health literacy and low numeracy are barriers to SDM, and the cultural backgrounds of some patients restrict them from making autonomous decisions. To better serve individuals, assessments and interventions should be selected after considering cultural factors including cultural preferences and norms [16]. In some non-Western cultures, the family plays a dominant role in decision making. The family is often an extension of the patient and assists the nurse in ensuring that the patient processes and understands information [6, 17]. Evidence suggests that people are influenced by their cultural background when making decisions regarding their health. These cultural values affect the manner in which people conduct themselves in the health care system and give patients a set of ethical priorities guiding their decisions regarding diagnosis and treatment [17].

Nursing staff account for the majority of the professional medical care team and are key members. They have many opportunities to participate in the SDM process with patients from various clinical departments. Truglio-Londrigan [6] indicated that studies on SDM experiences in nursing are limited. Although researchers have covered numerous medical and health care environments, no study has yet investigated the process or content of SDM. In particular, in most studies, the views of clinical nursing staff regarding SDM have been obtained from Western cultures [18]. In traditional Asian families (such as those in Taiwan), patients are more likely to play a silent role in the decision-making process because of traditional cultural pressure [17]. Thus, health care professionals should respect patients’ beliefs and values and what is important to them rather than what is important to the professionals themselves [19]. The family is crucial to patients in many aspects regardless of their cultural background. Moreover, the level of dominance shown by the family when a patient is involved in making crucial decisions can vary [17]. Therefore, given that cultural differences exist in the medical environment, exploring the SDM experiences of clinical nurses is necessary. This study explored the SDM experiences of clinical nurses to ensure that appropriate medical care is provided to patients and improve clinical care quality in the future.

## Methods

### Design and participants

A qualitative descriptive study aims to comprehensively summarize an event by using easy-to-understand sentences from the event [20]. Therefore, this study used a qualitative descriptive design to explore the course of SDM and the experiences of clinical nursing staff.

### Participants

In this study, intentional sampling was employed to recruit participants from a medical center in northern Taiwan from September 2018 to February 2019. Registered nurses who had worked in a hospital for a minimum of 1 year and who were willing to share their cultural experiences of being in clinical nursing care were included in this study. Nurses who had depression or other major illnesses (e.g., malignancies) were excluded. Depression is complex and often associated with other chronic conditions [21]. Nurses with depression are likely to be negatively affected by illness themselves, but their illness may also affect their coworkers and potentially the quality of the care they provide [22]. Therefore, these nurses were excluded from this study.

### Data collection

Interviews were arranged after obtaining the consent of research participants who met the inclusion criteria. The location of the interview was selected to ensure that interviewees could comfortably describe their experiences. In-depth, semistructured, face-to face interviews were conducted to collect data. Each interview began with general questions, followed by more specific questions. Some of the interview questions were as follows: “What do you know regarding the concept of shared decision making?” and “What do you think are the obstacles to implementing shared decision making?”. The audio recordings of the interviews ranged from 60 to 90 min in length and were immediately transcribed by a research assistant. In this study, data collection was continued until the data saturation point was reached. After interviewing 21 participants, we reached data saturation.

### Data analysis

All interviews were transcribed verbatim. Transcripts were first open coded word by word and line by line. Content analysis is usually begun in the early stage of data collection. The content analysis method reported by Zhang and Wildemuth [23] was used to analyze the interview data. This method consists of the following steps: preparing the data, defining the unit of analysis, developing categories and a coding scheme, testing the coding scheme on a sample text, coding all the text, assessing the coding consistency, drawing conclusions from the coded data, and reporting the methods and findings.

Qualitative content analysis goes beyond merely counting words or extracting objective content from texts; it involves examining meanings, themes, and patterns that may manifest or be latent in a particular text [23]. The content analysis in this qualitative research was performed as follows. First, the data were coded manually. All researchers participated in the data-coding process. After reading interview transcripts several times, crucial statements were identified and then the transcripts were compared across cases to determine similarities and differences in codes. Meaningful units were marked with codes, and a comparative analysis was performed to extract the primary code. Subsequently, the primary codes were according to differences and abstract the similarities in the categories and form the coding scheme. After testing the coding scheme on text samples, all text was coded. The data analysis started from the coding and was continued until the end of data collection.

### Rigor

Lincoln and Guba [24] developed four indicators to describe the suitability of qualitative research: dependability, confirmability, transferability, and credibility. These indicators were used to examine the rigor of our research results. Entire interviews were recorded, and the text analysis files were saved to ensure the dependability and confirmability of the data. This study used intentional sampling to determine the transferability of the research. The researcher interviewed each participant to obtain credible and promotional data regarding their experiences in the context of the medical care environment. In addition, the five researchers closely discussed and repeatedly examined the implications of the original data, determined which categories fit the original data, and provided operational definitions (peer debriefing) during the data analysis process to ensure credibility. After completion of the initial data analysis, three participants were asked to indicate whether the analysis results correctly described their experiences (member checks). These three participants responded that the results of this study were relevant to their experiences.

### Ethical considerations

 This study began recruiting participants after obtaining approval from the human testing institution of a medical center in northern Taiwan (Institutional Review Board: 18MMHIS123e). Before including a participant in this study, the researcher first explained the purpose and implementation steps of the research and proactively informed them that they had the right to withdraw from the study. The interview was conducted after obtaining signed informed consent from each participant.

## Results

A total of 21 participants who had been employed as nursing staff for an average of 18.7 years were included in this study. The most senior nurse had 37 years of work experience, whereas the most junior nurse had 3 years. In terms of work units, 9 (42.85%), 10 (47.61%), and 2 (9.5%) participants were from the departments of internal medicine, internal medicine intensive care, and pediatric intensive care, respectively. The findings of in-depth interviews and data analysis yielded three themes of clinical nurses’ experience in the SDM process: knowledge regarding SDM, trigger discussion and coordination, and respect of sociocultural factors. In SDM, nursing staff played the role of a “translator” by conveying the medical team’s findings and empirical information to the patient and their family members in an easy-to-understand manner. In addition, nursing staff were required to help family members make choices after listening to the thoughts of the patient and their family members.

### Knowledge regarding SDM

Knowledge regarding SDM led to health care professionals having a positive attitude and enhanced their willingness to practice SDM. Clinical nurses should possess knowledge regarding SDM. This theme consisted of the following categories: gaining relevant professional knowledge, reading and integrating evidence, and editing media regarding SDM.

#### Gaining relevant professional knowledge

To participate in the SDM process, nursing staff should be familiar with the concept of SDM in advance and then agree to it and be willing to implement it.

Interviewee M said the following:

...*the most basic [thing] for nursing staff is to know what SDM is. How did it start? Why did it start? What is its purpose? If the concept of SDM is not clear to nursing staff...Therefore, nursing staff should have good understanding regarding SDM before they can decide whether SDM will be helpful for the patient and they will be willing to implement it…*

During the SDM process, nurses must give a detailed explanation to the patient and their family members as well as respond to their various questions. Therefore, nurses must possess professional knowledge related to the theme of decision making. As stated by interviewee D, “...*I think professional ability is the most basic [ability]. You must be very clear regarding professional ability in the field because family members may ask various strange questions at any time and you must know how to respond to them...*”

Interviewee H indicated,

...*when we were in the process of SDM, the supervisor would arrange relevant on-the-job training...By using SDM auxiliary tools, we could focus more on patient care...Otherwise, sometimes, the nursing staff could not clearly answer questions relevant to the treatment of the patient. This is not okay.*

#### Reading and integrating evidence

SDM is relevant in the context of evidence-based practice. Evidence-based practice involves use of the best research with clinical expertise and patient values to facilitate decision making, leading to optimal clinical outcomes and quality of life. Therefore, reading and integrating evidence regarding SDM are critical for nursing staff in charge of SDM.

As interviewee J said,

...*Because we need to look for information to support our talk regarding SDM-related content, we must have the ability to read papers and then explain empirical concepts to the patient or family. Therefore, nursing staff must have the ability to construct empirical evidence.*

#### Editing media regarding SDM

Auxiliary tools, including patient decision aids (PDAs), are often required to enhance the understanding of patients and their family members regarding information provided during the SDM process. PDAs are structured tools, such as brochures and interactive online applications, that can aggregate available evidence related to a given decision and help patients clarify the relevant value of the decision [25]. Because the younger generation of Taiwan is not fluent in Taiwanese, it is necessary to have Taiwanese commentary videos available, particularly for older patients. However, all nursing staff are currently in charge of developing models and videos with limited funding.

One of the nursing staff who worked on editing, dubbing, quick response (QR) coding, and other related tasks said, “...*Making PDAs, such as videos, QR codes, and Google Forms, is not difficult for the nursing staff because this is what they usually do...*”

Some nursing staff also obtain resources to assist their own production of animations. Interviewee A stated, “...*when we are required to make SDM films, especially if we need an animation, we ask for the assistance of experts. The hospital has a unit that is involved in producing animations.*”

### Trigger discussion and coordination

The SDM process should involve the entire medical team. However, promoting SDM without the approval and participation of other medical staff in addition to the concerted efforts of nursing staff is challenging. This theme consisted of the following categories: forming a co-operative SDM team and trigger and coordination regarding SDM.

#### Forming a co-operative SDM team

SDM is a comprehensive concept based on the values and autonomy of patients, family members, doctors, and nursing staff. Because most decisions are related to medical treatment, doctors are the leaders of these decisions. However, some doctors have still not established the concept or habit of SDM.

As interviewee H said,

...*not every doctor has the knowledge or [has come to a] consensus regarding SDM. So, doctors may not use PDAs to explain the decision-making process to an individual patient, or doctors...do not use it in a way that the patient can understand, and whether they enter the spirit of SDM is doubtful.*

The success of SDM depends on constructing a favorable relationship during a clinical encounter that involves sharing information and supporting patients to deliberate and express their preferences and views during the SDM process.

 After participating in SDM with a doctor who agreed with the concept of SDM, interviewee A said,

*When promoting SDM, nursing staff co-operate with the chief doctor who supports SDM and influence other doctors through the chief doctor. Because the topic of SDM may be more strongly related to patient treatment, doctors and nursing staff should have a tacit understanding with each other [that] can help promote SDM.*

Interviewee C mentioned, “*Doctors are the main characters in promoting SDM and nursing staff assist doctors.*”

#### Trigger and coordination regarding SDM

The majority of interviewees believed that most problems related to SDM involved medical decisions. Therefore, the final decision makers are doctors, patients, and their family members. Nurses act as a communication bridge in the process. Nurses are required to communicate information to patients and their family members in an easy-to-understand manner after discussion with the doctor. In particular, the communication skills of nurses are most crucial when SDM must be implemented within a short time and when family members are under extreme pressure to discuss and make decisions in that limited amount of time, especially when the patient is critically ill.

As stated by interviewee B,

*When the patient or family members need to make a medical decision, I listen to their opinions first before searching for information. Sometimes, the attending physician does not have much time at the bedside, so I go over the analysis with the patient. If the patient says that he or she does not know which medical decision to make, I search for information again and discuss with the doctor again. [To participate in] SDM...[one] needs to have the ability to communicate...*

Handling advances and retreats during the communication process is essential. As interviewee F mentioned, “*Nursing staff should properly guide the patient and family to speak and communicate on the topic of SDM...to resonate with family members...then family members would be willing to talk. The talking skills and an ability to guide the talk are quite important.*”

In addition to conveying decision-related empirical information, nurses should guide and coordinate the concepts and expectations of both doctors and patients most of the time. Interviewee J indicated,

*During the SDM process, nursing staff need to coordinate or even connect with [people]. Like holding a family forum in the ward, the nursing staff should understand what content is unclear to family members and ask the doctor to explain. Also, the nursing staff should remind the family what they need to consider.*

The promotion of SDM should be based on a satisfactory nurse–patient relationship. In particular, when communicating with older patients, the ability to speak Taiwanese and other languages is essential. As interviewee B stated, “*Some elderly patients do not want young nursing staff to take care of them. They think that the scattered [Taiwanese] speaking...will affect the information they receive.*”

### Respect of sociocultural factors

The interviewees frequently noted that SDM requires patients and their family members to fully understand and consider what they want before making a decision. However, evidence suggests that people are influenced by their cultural background when making decisions regarding their health. This theme includes the following categories: patients’ values with respect to their cultural background, and the cultural differences of patients and families.

#### Patients’ values with respect to their cultural background

Cultural values influence the way in which people conduct themselves in the health care system and give patients a set of ethical priorities when making decisions regarding their diagnosis and treatment. Nurses should be patient and listen to the expectations of patients and their family members during the SDM process. To fully respect patients’ cultural values, nurses should respect the decision-making process they adopt even if it is collectivist and not based on equality within the family.

As stated by interviewee B, “*SDM needs to consider the experience and values of the patient.*”

In Taiwan, because of traditional cultural pressure, patients are more likely to play a silent role in the decision-making process.

Interviewee K mentioned that “*[There is a] need to understand the true thoughts of the patient. The patient will not immediately tell you what they are thinking...it takes a little bit of patience to listen.*”

#### Cultural differences of patients and families

The family is crucial to patients in many aspects regardless of their cultural background. Moreover, the level of dominance of the family can vary when a patient is involved in making crucial decisions. In some non-Western societies, the family plays a dominant role in decision making.

Interviewee C, who encountered a family member who refused to sign a “do not resuscitate” (DNR) form, said the following:

*The family members insist on their opinions and feel that they are not [displaying] filial piety if they sign the DNR. This [reflects] the personal values and background of the family member. The nursing staff can only directly explain to family members again…[and] respect the opinions of the family members in the end*.

Nursing staff must have the cultural sensitivity to demonstrate appropriate empathy and listening skills. In addition, nurses should be respectful of the wishes of family members even if they differ from those of the patient. Nursing staff must act as the spokesperson for the patient. Interviewee D said the following:

*The nursing staff should let the family members know the thoughts and wishes of the patient. When the patient has signed a consent or intention letter, the nursing staff should convey the patient’s wishes to the family members and doctors instead of agreeing with the final decision of the family members, with the decision being against the patient’s wishes.*

## Discussion

This study explored the experiences of clinical nurses participating in SDM. Studies have found that the SDM process is complicated for clinical nurses. The findings of this study yielded three themes of clinical nurses’ experiences in the SDM process: knowledge regarding SDM, trigger discussion and coordination, and respect of sociocultural factors.

Clinical decisions can be relatively simple (such as those involving general clinical treatment) or complex (such as those involving cancer treatment); be discrete (such as those involving the birth method) or involve continuous care management (such as when formulating chronic disease treatment and care plans); and can involve multiple stakeholders (such as the professional care team and caregivers of the patient) [26]. All interviewees in this study indicated that knowledge regarding SDM is extremely crucial in the SDM process. In addition, clinical nursing staff should understand professional concepts related to SDM. Our results are in accordance with those reported by Friesen-Storms et al. [9], who indicated that nursing staff having knowledge regarding SDM, skills, and a positive attitude can facilitate the SDM process. Moreover, our interviewees believed that they should first establish and be familiarized with the SDM concept, agree with it, and then be willing to implement it before conducting SDM. This result supports the finding of Mathijssen et al. [7], who indicated that improving medical professionals’ understanding of the SDM concept is the crucial first step for enhancing the implementation of SDM in clinical practice.

The interviewees in this study considered reading and integrating evidence and editing media regarding SDM to be critical abilities for implementing SDM continually. This result is in accordance with that reported by Tones et al. [27]; they found that when providing patients with various educational and interventional measures for effectively implementing SDM, it is necessary to collate the relevant literature and evidence and discuss the priorities of various behavioral changes with the patient and their family members in a language that they can easily understand. Subsequently, individualized patient health education aids can be developed to provide patient-centered and evidence-based health education to the patient and their family. Several studies have shown that nursing staff form the majority of a medical care team and are the team’s key members. To help patients make choices, nursing staff should not only use research evidence but also interpret that evidence or provide recommendations to meet the requirements of the patients in the SDM process. Therefore, nurses must be able to search for and integrate empirical data as well as understand basic concepts and principles related to SDM [9, 12]. The results of the present study revealed that nursing staff could help patients understand the disease, clinical progress, and treatment options by using information software during the implementation of SDM. Therefore, the nursing staff believed that having the basic ability to edit media was indispensable. This result is in accordance with that reported by Friesen-Storms et al. [9], who found that providing nursing staff with SDM training, such as training in media editing and the creation of PDAs, and guidance in developing a patient-centered attitude could significantly improve the implementation of SDM by nursing staff.

SDM is a framework in which health professionals and patients co-operate to make decisions during implementation of a series of medical procedures [28]. Satisfactory clinical communication skills are crucial in nursing staff for establishing effective SDM [9]. The participants in this study all agreed on the importance of trigger discussion and coordination. The final decision makers in SDM are doctors, patients, and family members. However, nurses still account for the majority of medical care professionals [9, 12]. The interviewees in this study indicated that the attending physician sometimes did not have sufficient time to participate at the bedside while performing clinical SDM, thus limiting the implementation of SDM. This finding is similar to that of an Asian study conducted by Lin et al. [29], who reported that most patients felt that health professionals, even if they agreed to implement SDM, had limited resources available to provide adequate information or support to patients in making decisions. This result is in accordance with that of Mathijssen et al. [7], who determined that time limitation was an issue for during implementation of SDM in clinics. In addition, the present finding indicated that nursing staff play the crucial role of a communication bridge in the implementation of SDM.

The interviewees indicated that promoting SDM without the approval and participation of the decision-making leader (doctor) is challenging. This is another crucial finding of the present study. Therefore, forming a co-operative SDM team is an essential factor for promoting SDM. This result is in agreement with those of several studies. Hofstede et al. [30] conducted a study on SDM for patients with rheumatology and indicated that although the medical staff all had the same knowledge, attitude, and experiences regarding SDM in rheumatology, lack of co-operation between professional groups was an essential obstacle to implementation of SDM. Patients may receive conflicting information from different medical professionals. Therefore, SDM requires effective communication between medical professionals to provide structured information to patients [7]. Our interviewees indicated that the theme of SDM was related to the treatment of the patient, doctors played a primary role in implementing SDM, and nursing staff assisted doctors in promoting SDM. These findings are similar to those of other studies [7, 29]. Mathijssen et al. [7] investigated the SDM-related knowledge, attitude, and experiences of 147 medical staff and revealed that under SDM, decisions regarding diagnostic tests were based on doctors’ input because making decisions regarding patients’ disease treatment and diagnosis was not the task or responsibility of nursing staff. Lin et al. [29] investigated patients’ perspectives on SDM in Taiwan; they discovered that patients had a desire to be involved and felt that adequate information exchange would be a necessary step toward collaboration or sharing treatment-related decisions with clinicians. Most clinics have used interprofessional practice to improve the quality of care in recent years. Therefore, the subject of co-operation among interprofessional teams for the implementation of SDM has also been valued. Dawn and Legare [31] indicated that oncology nursing staff were the key members of interprofessional practice in terms of exerting influence, particularly when patients had to make a decision regarding prevention, screening, or treatment options during the SDM process. The importance of the role of nursing staff in SDM could also be observed in interprofessional practice.

In Taiwan, because of traditional cultural pressure, patients are more likely to play a silent role in the decision-making process than in other countries. Lin et al. [29] reported that in submissive Asian cultures, SDM implementation can be more challenging. The importance of nurses’ respect of sociocultural factors during the SDM process was a crucial finding of this study. Crucial to SDM implementation is the effective participation of patients. Because different patients have different backgrounds, characteristics, and value preferences, patients may make various choices and value judgments when it comes to clinical decisions [32]. Several studies have shown that the cultural factors of patients should be considered when performing SDM [18, 29, 33, 34]. Patients have independent autonomic and informed rights as well as the right to insist on care and choose their treatment plan. Unlike other medical care measures that can directly improve the symptoms of patients through care behavior, SDM may exert a positive effect on the future medical treatment of patients, ultimately leading to better health outcomes [35]. The present study indicated that nursing staff should listen to the requirements of patients and their family members who expect SDM, and patients and their family members should fully consider what they want before making a decision. When we understand this culture, the results of the study will become clear because they reflect the traditional means of decision making. This result is in accordance with that of Mathijssen et al. [7], who found that understanding the willingness and degree to which patients wish to participate in decision making is crucial for medical professionals. Sims-Gould and Martin-Matthews [36] postulated that working with patients and their family members in an interconnected, bidirectional manner and recognizing and supporting cultural ideas, values, and beliefs can help the patients and family members in becoming co-producers in health and thus aid the implementation of SDM. Thus, these findings indicated that respect and cultural sensitivity are crucial factors in the SDM process.

Because the participants in this study were chosen from nursing staff in a medical center in northern Taiwan, the results cannot be applied to all nursing staff. In addition, the self-responses of medical and nursing staff regarding their attitude and experiences related to SDM (such as “In what situation do you think it is suitable to use SDM?”) may have been affected by their definition of SDM. Moreover, nursing staff with a positive attitude toward SDM may have been more inclined to participate in this study. Therefore, the probability of bias in sample selection cannot be ruled out. Future studies should expand their sample sources to explore the SDM experiences of multiple nursing staff members and thus provide a more complete reference base for relevant patient care.

## Conclusion

This study explored the experiences of clinical nursing staff participating in SDM by conducting in-depth interviews. The results yielded three themes of the implementation of the SDM process for clinical nurses: knowledge regarding SDM, trigger discussion and coordination, and respect of sociocultural factors. The promotion of SDM can help nursing staff more deeply explore the thoughts and expectations of patients and their family members as well as confirm the direction of care.

## Supplementary Information

**Additional file 1.** Shared clinical decision-making experiences in nursing: A qualitative study*, * Interview guide *.

## Data Availability

The datasets used and analysed during the current study are available from the corresponding authors on reasonable request.

## Abbreviations

SDM: Shared Decision Making
DNR: Do Not Resuscitate
PDAs: Patient Decision Aids
IPP: Interprofessional practice

## References

[1] Stiggelbout AM, Pieterse AH, De Haes JC (2015). Shared decision making: Concepts, evidence, and practice. Patient Educ Couns.

[2] Golanowski M, Beaudry D, Kurz L, Laffey WJ, Hook ML (2007). Interdisciplinary shared decision making: taking shared governance to the next level. Nurs Adm Q.

[3] Sieck CJ, Johansen M, Stewart J (2016). Inter-professional shared decision making – increasing the “shared” in shared decision making. Int J Healthc.

[4] Elwyn G, Frosch D, Thomson R, Joseph-Williams N, Lloyd A, Kinnersley P, Cording E, Tomson D, Dodd C, Rollnick S, Edwards A, Barry M (2012). Shared decision making: a model for clinical practice. J Gen Intern Med.

[5] Makoul G, Clayman ML (2006). An integrative model of shared decision making in medical encounters. Patient Educ Couns.

[6] Truglio-Londrigan M (2013). Shared decision-making in home-care from the nurse’s perspective: sitting at the kitchen table– a qualitative descriptive study. J Clin Nurs.

[7] Mathijssen EGE, van den Bemt BJF, Wielsma S, van den Hoogen FHJ, Vriezekolk JE (2020). Exploring healthcare professionals’ knowledge, attitudes and experiences of shared decision making in rheumatology. RMD Open.

[8] Brembo EA, Eide H, Lauritzen M, van Dulmen S, Kasper J (2020). Building ground for didactics in a patient decision aid for hip osteoarthritis. Exploring patient-related barriers and facilitators towards shared decision-making. Patient Educ Couns.

[9] Friesen-Storms J, Bours G, Weijden T, Beurskens A. Shared decision making in chronic care in the context of evidence based practice in nursing. Int. J. Nurs. Stud. 2015;52 (2015): 393–402. 10.1016/j.ijnurstu.2014.06.012 25059684

[10] Karlsen M, Happ MB, Finset A, Heggdal K, Heyn LG (2020). Patient involvement in micro-decisions in intensive care. Patient Educ Couns.

[11] Murray K, Yasso S, Schomburg R, Terhune M, Beidelschies M, Bowers D, Goodyear-Bruch C (2016). Journey of excellence: Implementing a shared decision-making model. Am J Nurs.

[12] Ervin K, Blackberry I, Haines H (2017). Developing a taxonomy and mapping concepts of shared decision making to improve clinicians understanding. NCOAJ.

[13] Tariman JD, Mehmeti E, Spawn N, McCarter SP, Bishop-Royse J, Garcia I, Hartle L, Szubski K (2016). Oncology nursing and shared decision making for cancer treatment. Clin J Oncol Nurs.

[14] Jo KH, An GJ, Lee HS. Health care professional factors influencing shared medical decision making in Korea. SAGE Open 2015; 1–8. 2158244015614608.

[15] Charles C, Gafni A, Whelan T (1997). Shared decision-making in the medical encounter: what does it mean? (or it takes at least two to tango). Soc Sci Med.

[16] Fong EH, Catagnus RM, Brodhead MT, Quigley S, Field S (2016). Developing the Cultural Awareness Skills of Behavior Analysts. Behav Anal Pract.

[17] Gilbar R, Miola J (2015). One size fits all? on patient autonoby, medical decision-making, and the impact of culture. Med Law Rev.

[18] Obeidat RF, Homish GG, Lally RM (2013). Shared decision making among individuals with cancer in non-Western cultures: a literature review. Oncol Nurs Forum.

[19] Alex J, Ramjan L, Salamonson Y, Ferguson C (2020). Nurses as key advocates of self-care approaches to chronic disease management. Contemp Nurse.

[20] Sandelowski M (2000). Whatever happened to qualitative description?. Res. Nurs. Health.

[21] Brandford AA, Reed DB (2016). Depression in registered nurses: A state of the science. Workplace Health Saf.

[22] Letvak S, Ruhm CJ, McCoy T (2012). Depression in hospital employed nurses. Clin Nurse Spec.

[23] Zhang Y, Wildemuth BM. “Qualitative Analysis of Content,” In: B. M. Wildemuth, Ed., Applications of social research methods to questions in information and library science, Libraries Unlimited, 2009. pp. 1–12. https://www.semanticscholar.org/paper/Qualitative-Analysis-of-Content-by-Zhang-Wildemuth/b269343ab82ba8b7a343b893815a0bae6472fcca.

[24] Lincoln YS, Guba EG (1989). Fourth generation evaluation.

[25] Légaré F, Witteman HO (2013). Shared decision making: examining key elements and barriers to adoption into routine clinical practice. Health Aff.

[26] Tay E, Vlaev I, Massaro S. (2017). Toward a Behavioral Model of Shared Decision Making, Academy of Management Annual Meeting Proceedings; 2017(1):13986. 10.5465/AMBPP.2017.13986abstract.

[27] Tones K, Tilford S (1994). Health education: effectiveness, efficiency and equity.

[28] Astbury R, Shepherd A, Cheyne H (2017). Working in partnership: the application of shared decision making to health visitor practice. J Clin Nurs.

[29] Lin CY, Renwick L, Lovell K. Patients’ perspectives on shared decision making in secondary mental healthcare in Taiwan: A qualitative study. Patient Educ. Couns. 2020; 103(12)2020, 2565–2570, 10.1016/j.pec.2020.05.030. 10.1016/j.pec.2020.05.030 32487469

[30] Hofstede SN, Marang-van de Mheen PJ, Wentink MM, Stiggelbout AM, Vleggeert-Lankamp CL, Vliet Vlieland TP, van Bodegom-Vos L, DISC study group (2013). Barriers and facilitators to implement shared decision making in multidisciplinary sciatica care: A qualitative study. Implementation Sci.

[31] Dawn S, Légaré F (2015). Engaging patients using an interprofessional approach to shared decision making. Can Oncol Nurs J.

[32] Montori VM, Brito JP, Murad MH (2013). The optimal practice of evidence-based medicine: Incorporating patient preferences in practice guidelines. JAMA.

[33] Hawley ST, Morris AM (2017). Cultural challenges to engaging patients in shared decision making. Patient Educ Couns.

[34] Mữller E, Hahlweg P, Scholl I (2016). What do stakeholders need to implement shared decision making in routine cancer care? A qualitative needs assessment. Acta Oncol.

[35] Oshima Lee E, Emanuel EJ (2013). Shared decision making to improve care and reduce costs. N Engl J Med.

[36] Sims-Gould J, Martin-Matthews A (2010). We share the care: family caregivers’ experiences of their older relative receiving home support services. Health Soc Care Community.

