# Supplementary material for: Shared clinical decision-making experiences in nursing: a qualitative study
Source: BMC Nurs. 2021 Jun 1;20:85. doi: 10.1186/s12912-021-00597-0 (PMC8167946; doi:10.1186/s12912-021-00597-0)
Supplement: Supplementary file 1 — Additional file 1. Shared clinical decision-making experiences in nursing: A qualitative study*, * Interview guide *. [file 12912_2021_597_MOESM1_ESM.pdf]

Interview guide

- What do you know regarding the concept of shared decision making?
- In your opinion, who should make treatment decisions?
- What do you think is the use of the concept of shared decision-making for the clinical care plan of individual patients?
- Who usually makes treatment decisions clinically?
- How do you use shared decision-making for individual patients in your daily care?
- What do you think of the role and function of nursing staff in the decision-making sharing process?
- What abilities do you think nurses need to possess or improve to achieve shared decision-making?
- Please think about the most recent treatment meeting you attended, at which a treatment decision was made.
- What do you think are the obstacles to implementing shared decision making?
